# Conserving Biodiversity Efficiently: What to Do, Where, and When

**DOI:** 10.1371/journal.pbio.0050223

**Published:** 2007-08-21

**Authors:** Kerrie A Wilson, Emma C Underwood, Scott A Morrison, Kirk R Klausmeyer, William W Murdoch, Belinda Reyers, Grant Wardell-Johnson, Pablo A Marquet, Phil W Rundel, Marissa F McBride, Robert L Pressey, Michael Bode, Jon M Hoekstra, Sandy Andelman, Michael Looker, Carlo Rondinini, Peter Kareiva, M. Rebecca Shaw, Hugh P Possingham

**Affiliations:** 1 The Ecology Centre, School of Integrative Biology, University of Queensland, St. Lucia, Queensland, Australia; 2 Department of Environmental Science and Policy, University of California Davis, Davis, California, United States of America; 3 The Nature Conservancy, San Diego, California, United States of America; 4 The Nature Conservancy, San Francisco, California, United States of America; 5 Department of Ecology, Evolution and Marine Biology, University of California Santa Barbara, Santa Barbara, California, United States of America; 6 Natural Resources and the Environment, Council for Scientific and Industrial Research, Stellenbosch, South Africa; 7 School of Natural and Rural Systems Management, University of Queensland, Gatton, Queensland, Australia; 8 Center for Advanced Studies in Ecology and Biodiversty, Santiago, Chile; 9 Departamento de Ecología, Facultad de Ciencias Biológicas, Pontificia Universidad Católica de Chile, Santiago, Chile; 10 Instituto de Ecología y Biodiversidad, Santiago, Chile; 11 National Center for Ecological Analysis and Synthesis, University of California Santa Barbara, Santa Barbara, California, United States of America; 12 Department of Ecology and Environmental Biology, University of California Los Angeles, Los Angeles, California, United States of America; 13 Center for Embedded Networked Sensing, University of California Los Angeles, Los Angeles, California, United States of America; 14 School of Botany, University of Melbourne, Parkville, Melbourne, Australia; 15 The Nature Conservancy, Seattle, Washington, United States of America; 16 Tropical Ecology, Assessment and Monitoring Program, Conservation International, Washington, D.C., United States of America; 17 The Nature Conservancy, Australia Program, Carlton, Victoria, Australia; 18 Dipartimenti di Biologia Animale e dell'Uomo, Università la Sapienza, Rome, Italy; Imperial College London, United Kingdom

## Abstract

Conservation priority-setting schemes have not yet combined geographic priorities with a framework that can guide the allocation of funds among alternate conservation actions that address specific threats. We develop such a framework, and apply it to 17 of the world's 39 Mediterranean ecoregions. This framework offers an improvement over approaches that only focus on land purchase or species richness and do not account for threats. We discover that one could protect many more plant and vertebrate species by investing in a sequence of conservation actions targeted towards specific threats, such as invasive species control, land acquisition, and off-reserve management, than by relying solely on acquiring land for protected areas. Applying this new framework will ensure investment in actions that provide the most cost-effective outcomes for biodiversity conservation. This will help to minimise the misallocation of scarce conservation resources.

## Author Summary

Given limited funds for biodiversity conservation, we need to carefully prioritise where funds are spent. Various schemes have been developed to set priorities for conservation spending among different countries and regions. However, there is no framework for guiding the allocation of funds among alternative conservation actions that address specific threats. Here, we develop such a framework, and apply it to 17 of the world's 39 Mediterranean-climate ecoregions. We discover that one could protect many more plant and vertebrate species by investing in a sequence of conservation actions targeted towards specific threats, such as invasive species control and fire management, rather than by relying solely on acquiring land for protected areas. Applying this new framework will ensure investment in actions that provide the most cost-effective outcomes for biodiversity conservation.

## Abstract 3

A new framework applied to 17 of the world's Mediterranean ecoregions reveals that investing in a sequence of conservation actions targeted towards specific threats will protect more species than just acquiring land for protected areas.

## Introduction

Many sophisticated approaches exist for identifying priority areas for conservation at a global scale. These “biodiversity hotspots” or “crisis ecoregions” are typically identified using data on endemic species richness, total biodiversity, and past habitat conversion [1–3]. With few exceptions, these approaches neglect economic costs and provide a static assessment of conservation priorities. They therefore cannot provide guidance on how funds should be distributed between regions, nor can they inform when the funds should be spent. Recent theoretical advances incorporate economic considerations and landscape dynamics into priority-setting, and provide an analytical framework for deciding where, when, and how much money should be invested for biodiversity conservation [4–8].

While these theoretical advances incorporate economic considerations, they treat land acquisition, or the creation of protected areas, as a surrogate for the broader suite of actions available to protect biodiversity. Conservation practitioners routinely invest in a diverse array of activities such as fire management, invasive species control, and revegetation, with the aim of enhancing or sustaining biodiversity. In many places land acquisition is not feasible, and neither appropriate nor affordable. In addition, the spatial extent of many threats is usually greater than the area of land that can be acquired. A framework is urgently needed that can support the more sophisticated funding allocation decisions required from conservation practitioners. Such a framework could help to allocate limited conservation funds to threat-specific conservation actions in areas where they are likely to achieve the greatest potential biodiversity benefit.

**Figure 1:**
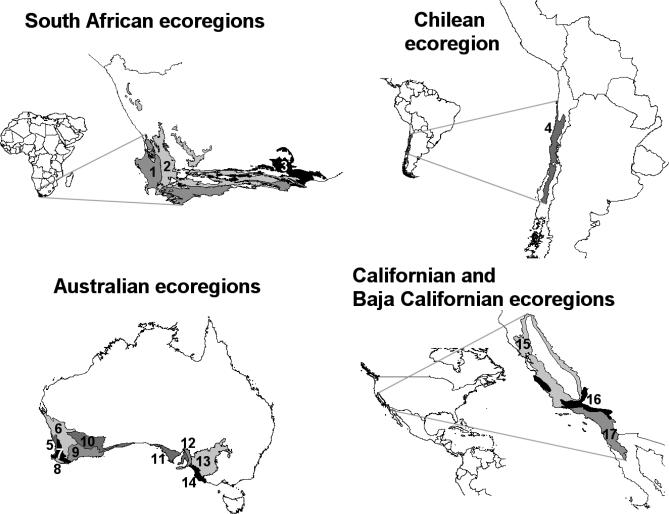
The Mediterranean Ecoregions of South Africa, Chile, Australia, and California/Baja California Lowland fynbos and renosterveld (1), montane fynbos and renosterveld (2), Albany thickets (3), Chilean matorral (4), Swan Coastal Plain scrub and woodlands (5), Southwest Australia savanna (6), Southwest Australia woodlands (7), Jarrah-Karri forest and shrublands (8), Esperance mallee (9), Coolgardie woodlands (10), Eyre and York mallee (11), Mount Lofty woodlands (12), Murray-Darling woodlands and mallee (13), Naracoorte woodlands (14), interior chaparral and woodlands (15), montane chaparral and woodlands (16), and coastal sage scrub and chaparral (17).

Here, we develop an action- and area-specific framework for conservation investment and illustrate its application using Mediterranean-type habitats (Figure 1). Mediterranean ecoregions boast exceptional species diversity but are poorly protected, highly degraded, and exposed to multiple persistent threats [9–13]. Consequently, they have been ranked among the world's highest conservation priorities [3,14,15]. How might funds be allocated to conserve Mediterranean ecoregions in the most cost-effective way?

**Figure 2:**
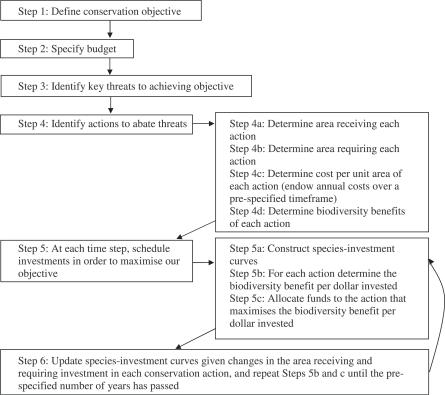
Decision Steps Involved in the Conservation Investment Framework

To apply our framework (Figure 2 and see Materials and Methods) we require an explicit statement of the overall conservation objective and the budget (steps 1 and 2 of Figure 2), and an understanding of the threats operating in each ecoregion and the potential conservation actions to abate them (steps 3 and 4). Our objective is to maximise the total number of species (vascular plants and vertebrates combined) conserved across these ecoregions, through strategic investment in a suite of conservation actions, given a fixed annual budget. The amount of money allocated annually to each conservation action in each ecoregion depends on the area of land currently receiving and requiring the action, the cost of the action per unit area, and the biodiversity benefited by the investment (the number of plant and vertebrate species predicted to persist in an ecoregion after investment in a conservation action; see Materials and Methods).

Our aim is to develop investment schedules for Mediterranean ecoregions that reflect the relative returns from investing in different conservation actions in order to maximise our objective (step 5 of Figure 2). We deliver investment priorities that change through time depending on the cumulative impacts of investments (step 6). While we address a global-scale problem, our framework and analytical approach is also applicable at national and regional scales.

## Materials and Methods

We apply our framework to the 17 (of 39) terrestrial Mediterranean ecoregions for which data are most readily accessible. This subset of Mediterranean ecoregions covers parts of Australia (ten ecoregions; Table 1), Chile (one ecoregion; Table 2), South Africa (three ecoregions; Table 3), and California and Baja California (three ecoregions; Table 4) (Figure 1). Although we recognise that alternative delineations of Mediterranean habitats are available, we employ the delineations provided by the World Wildlife Fund, given their utility for global-scale analyses.

**Table 1:**
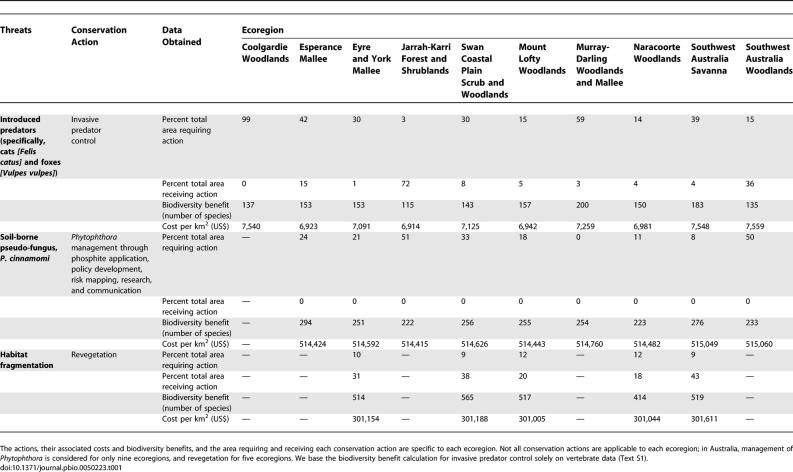
Threats and Conservation Actions Analysed for the Ten Australian Mediterranean Ecoregions

**Table 2:**
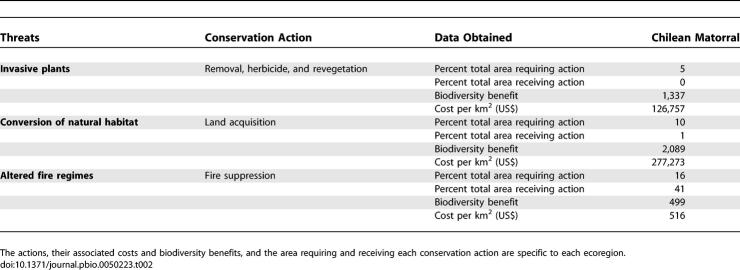
Threats and Conservation Actions Analysed for the Chilean Mediterranean Ecoregion

**Table 3:**
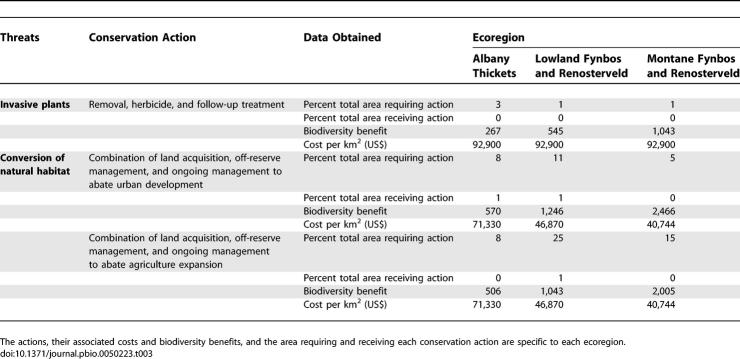
Threats and Conservation Actions Analysed for the South African Mediterranean Ecoregions

**Table 4:**
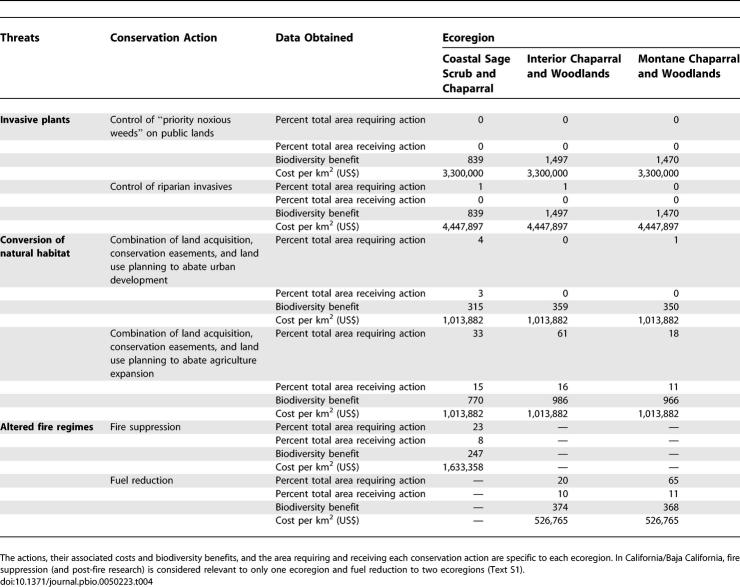
Threats and Conservation Actions Analysed for the Californian/Baja Californian Mediterranean Ecoregions

Through consultation with regional experts, we identify the key threats in each ecoregion to achieving our objective of maximising the total number of species (in our case, vascular plants and vertebrates combined) conserved given an annual budget of US$100 million (steps 1–3 of Figure 2). We also identified the actions undertaken to abate these threats (step 4). Hereafter we term each ecoregion–conservation action combination an “ecoaction”. We assume that the impact of each ecoaction is independent. Through a combination of expert input, literature review, and analysis of regional datasets in geographic information systems we determine the areas requiring, and already receiving, each ecoaction and estimate the cost associated with its implementation (steps 4a–4c of Figure 2; Text S1).

While the costs incurred for some conservation actions, such as land acquisition or revegetation, are one-time costs, the costs of other actions, such as invasive predator control, are incurred annually. To convert the latter to one-time costs, we endow the annual cost over 20 y (unless otherwise stated), after which further funds are required for these conservation actions to continue. We determine the endowed value by calculating the net present value over the timeframe of interest, assuming an inflation rate of 3.2% and discount rate of 6.04%. This discount rate is equivalent to a 10-y US government bond rate, and the inflation rate represents that of the US dollar in 2005. We account for the costs of ongoing management for ecoactions that involve land acquisition and for the costs of establishing agreements with private landholders (if such investments are considered necessary for an ecoaction to proceed or to be long-lasting; Text S1). The cost of each ecoaction is based on the perceived expenditure required for successful interventions, and we therefore assume that investment in each ecoaction will prevent the local extinction of species at risk from the relevant threat.

In this paper, the number of species benefited by each ecoaction—its “biodiversity benefit”—is the number of plant and vertebrate species predicted to persist in an ecoregion after investment in the ecoaction (step 4d of Figure 2). If appropriate data were available for each ecoaction, we could modify the predicted biodiversity benefit by the likelihood that the ecoaction will succeed in abating the relevant threat. To operationalise our approach we need a functional form for the relationship between investment in an ecoaction and its biodiversity benefit. Every investment shows diminishing returns and therefore we assume that the marginal benefit of investment in a particular ecoaction decreases as the size of the investment increases. We represent diminishing returns using the functional form of the species–area relationship, where the total number of species *(S)* present in area *A* is a power-law function of that area [16]:

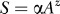
We calculate the constant α by dividing the total number of species in the ecoregion by the estimated area of original habitat, after raising this area to the power of *z* (see Text S2). In the baseline scenario we assign *z* a value of 0.2, a typical value for terrestrial, non-island regions [16].

We therefore assume that the incremental number of species protected with a given increase in area protected follows the form of a standard species–area curve. When we account for the cost of each ecoaction, we simply replace the area protected by the cost of protecting the equivalent area (to generate a species–investment curve; step 5a of Figure 2). This relationship is straightforward for habitat protection or restoration, but requires further thought for the diverse array of conservation actions considered here.

The adaptation of species–area curves to conservation actions other than reserving or restoring land is based on the premise that investment in these actions will also exhibit diminishing returns. The major refinements required are that the area of “protected” habitat is the area of investment in each ecoaction (each with a pre-specified cost) and the number of species protected is threat-specific. Since we currently do not have an ecological basis for an alternative parameterisation of this relationship for the range of ecoactions considered here, we evaluate the sensitivity of the allocation schedules to the value of *z*. We choose *z* randomly from a uniform distribution (between 0.1 and 0.4, *n =* 30) to reflect the uncertainty about the relationship between the number of species protected and the amount of money invested in each ecoaction, specifically, the rate at which the returns from investment diminish.

To determine the biodiversity benefit of an ecoaction that abates a specific threat we consider only those species impacted by that threat. We calculate the number of “at risk” species by determining the proportion of plant and vertebrate species regarded as threatened by each type of threat (using the World Conservation Union [IUCN] Red List for each country [17] and excluding those species that are of least concern or data deficient), and multiply this proportion by the total number of plant and vertebrate species occurring in each ecoregion [18,19]. Thus we assume that the proportion of species that would be protected by investment in a particular ecoaction is the same as the proportion of IUCN-listed species identified nationally as being at risk from the relevant threat, and that the species in this subset benefit equally from an investment. For invasive predator control in Australia, we limit the biodiversity benefit calculation to just vertebrates by restricting the IUCN search to vertebrate species and multiplying this proportion by the number of vertebrate species occurring in each ecoregion.

Obtaining an optimal allocation schedule through time amongst such a large number of ecoactions is computationally intractable [4,5], so we adopt a “rule of thumb”, or heuristic, to approximate the optimal investment schedule. This heuristic, which we term “maximise short-term gain”, directs funds each year to ecoactions that provide the greatest short-term increase in biodiversity benefit per dollar invested (steps 5b and 5c of Figure 2). Using this heuristic we generate an investment schedule over 20 y, given a fixed annual budget of US$100 million (step 6 of Figure 2). We assess the sensitivity of the investment schedule to the budget size by repeating the analysis with the annual budget reduced to US$10 million.

We use the Spearman coefficient of rank correlation to compare the priority rankings based on the ecoaction-specific framework to those based on a ranking of vertebrate species richness [20]. Because of lack of independence, we test significance against the distributions of Spearman values derived from 100,000 random pairings of *X* and *Y* variables. The null hypothesis is that the observed coefficient is zero, or the distribution of *Y* is the same for all values of *X* [21].

We also compare the ecoaction-specific framework to a simplified model of conservation that focuses only on land acquisition for the creation of protected areas. In this analysis, we estimate the cost of land acquisition using a statistical model (Table S1) and the area requiring acquisition as the area of natural habitat that is currently unprotected (IUCN status I–IV). We estimate the biodiversity benefit of this action as the total number of plant and vertebrate species. Therefore, while the biodiversity benefit under the ecoaction-specific framework is a proportion of the total species richness (those threatened by each threat type), the biodiversity benefit under the acquisition-only framework is the total richness of vertebrate and plant species. As with the ecoaction approach, we assume diminishing returns with cumulative investment and model this relationship using species–investment curves. For the acquisition-only approach we rescale the annual budget to US$148 million dollars (from US$100 million), since the overall cost of achieving our objective under this scenario is 48% greater.

## Results

Different ecoregions have different mixtures of threats and candidate conservation actions. In total, we evaluated 51 ecoregion–conservation action combinations (denoted “ecoactions”; Tables 1–4) across 17 Mediterranean ecoregions (Figure 1). Using species–investment curves for each of the 51 ecoactions and the “maximise short-term gain” heuristic (see Materials and Methods), we obtained investment schedules based on an annual budget of US$100 million. These schedules reflect shifts in the allocation of funds as the return from investing in each ecoaction diminishes. The investment schedules are determined by the interplay of three main factors: (1) the relationship between the additional area invested in each ecoaction and the biodiversity benefit; (2) the cost of this investment; and (3) the existing level of investment.

### A Regional Example

**Figure 3:**
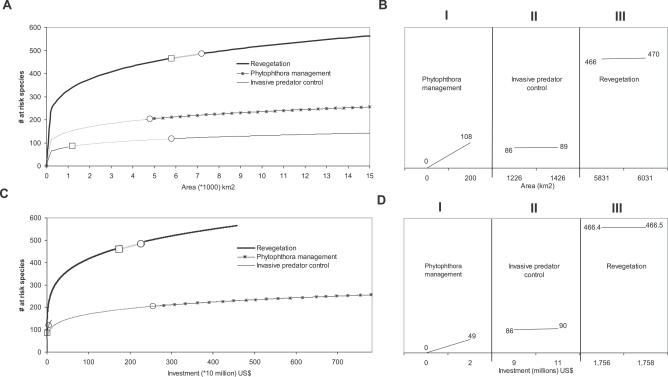
Species–Area Curves and Species–Investment Curves for Three Conservation Actions The three actions portrayed are *Phytophthora* management, invasive predator control, and revegetation in the Swan Coastal Plain scrub and woodlands ecoregion of Australia. In (A) and (C), the area of conservation interest is illustrated by the curve to the left of the circles, comprising the area already receiving the action (denoted by the solid lines between the origin and the squares) and the area requiring the action (denoted by the dashed lines between the squares and the circles). Three categories of species are represented within the tail of the curves, beyond the area of conservation interest (that is, to the right of the circles). The species within these categories are not necessarily mutually exclusive. The first contains at risk species whose populations also occur in habitat outside the area of conservation interest for a particular threat. These species do not rely fully on the conservation action to persist in the ecoregion. The second category also reflects the uncertainty about the distributions of species, as these species do not actually occur within the area of conservation interest. The third category represents part of the extinction debt due to past habitat loss and contains at risk species that are unlikely to persist in the ecoregion over the long term, regardless of the conservation action. The number of species in each of the three groups cannot be determined in this example, since our method of estimating biodiversity benefit does not allow the individual species or their geographic distributions to be identified (Text S2). (A) Species–area curves. (B) The number of at risk species protected after an additional investment of 200 km^2^ in each conservation action (the panels are close-ups of the curves in [A] and represent the benefit of the additional investment from the area already receiving the action). (C) Species–investment curves, where the area invested in each action is replaced by the cost of the ecoaction. (D) The number of at risk species protected before and after an additional investment of US$2 million in each conservation action (the panels are close-ups of the curves in [C] and represent the benefit of the additional investment in the area already receiving the action).

We illustrate how these three factors interact within our conservation investment framework (steps 4–6 of Figure 2) using a regional-scale case study from the Swan Coastal Plain scrub and woodlands ecoregion of Australia (Figure 3). The curve to the left of the circles indicates the total area of conservation interest (indicated by the squares), that is, the area already receiving the actions (step 4a), and the area requiring them (step 4b). For example, the area of interest for invasive predator control in this ecoregion is about 5,832 km^2^ (see Text S1). The curve to the right of the circles acknowledges that the persistence of species depends on past changes to habitat and that knowledge of the distribution of species is uncertain (see Text S2).

We estimated the original extent of habitat in this ecoregion to be approximately 15,210 km^2^ and that this area supported a total of 565 vertebrate and plant species now at risk due to habitat fragmentation, 256 plant and vertebrate species now at risk from a soil-borne pseudo-fungus, *Phytophthora cinnamomi,* and 143 vertebrate species now at risk due to invasive predators (step 4d of Figure 2). In each case, the total number of species estimated to be at risk is represented by the right-hand endpoint of the species–area curves (Figure 3A).

Assuming the costs of undertaking the different actions is the same, we determined that conducting invasive predator control or revegetation over an additional 200 km^2^ in the Swan Coastal Plain ecoregion will potentially protect three and four species, respectively (panels II and III of Figure 3B). Conducting *Phytophthora* management over the same area has the potential to protect 108 species because the area of conservation interest lies in the steepest part of the species–area curve (Text S1; Table 1; panel I of Figure 3B).

When we modelled the relationship between the biodiversity benefit and dollars invested using species–investment curves (step 5a of Figure 2; Figure 3C), we found that the cost-effectiveness of each action varies widely (step 5b). Revegetation in the Swan Coastal Plain ecoregion costs US$301,118 per square kilometre, *Phytophthora* management costs US$514,626 per square kilometre, and invasive predator control costs US$7,125 per square kilometre (Table 1). *Phytophthora* management was still the most cost-effective action: US$2 million spent on this action will potentially protect 49 species, although the potential benefit reduces rapidly with cumulative investment (panel I of Figure 3D). An initial expenditure of US$2 million on invasive predator control in the Swan Coastal Plain ecoregion has the potential to protect four species, whereas there is negligible benefit from spending US$2 million on revegetation (panels II and III of Figure 3D). The comparatively greater marginal returns from investing in invasive predator control are due to its low cost, despite the fact that the direct biodiversity benefit for this action is restricted to vertebrates.

Based on this analysis, initial investment within the Swan Coastal Plain scrub and woodlands ecoregion is prioritised to *Phythopthora* management (step 5c of Figure 2). The species–investment curves are then updated given changes in the area receiving the conservation action and the area requiring the conservation action (step 6). In the next time step, the budget is allocated to the conservation action that now maximises the biodiversity benefit per dollar invested. This regional case study therefore illustrates how the species–investment curves are constructed, and how the actions are prioritised for investment at each time step based on their cost and biodiversity benefits, and the current level of investment in each conservation action.

### Global Investment Priorities

In applying the conservation investment framework at the global scale we encompass a greater mix of threats and candidate conservation actions. Across all 17 ecoregions, only 24 ecoactions (of the 51 possible) received investment in the model during the first 5 y. During this time, most funds were allocated to land protection and management (through land acquisition, off-reserve management, and on-going management) in the three South African ecoregions (66% of the total budget, six ecoactions in total; Table 5). Much of the remaining funds were allocated to invasive plant control in the Chilean ecoregion, the three South African ecoregions, and one of the Californian/Baja Californian ecoregions (24% of the total budget; five ecoactions in total; Table 5). These conservation actions yielded the greatest marginal return on investment over 5 y because the potential biodiversity benefit is high and the costs are comparatively low. Over 5 y the greatest amount of money (21% of the total budget) is allocated to land protection and management (through land acquisition, off-reserve management, and on-going management) to abate agricultural conversion in the montane fynbos and renosterveld ecoregion of South Africa. This broad ecoregion contains a large area of arable land that is unconverted but largely unprotected. Furthermore, the potential biodiversity benefit of abating agricultural conversion in this region is high, while the cost of this ecoaction is comparatively low (Table 3).

**Table 5:**
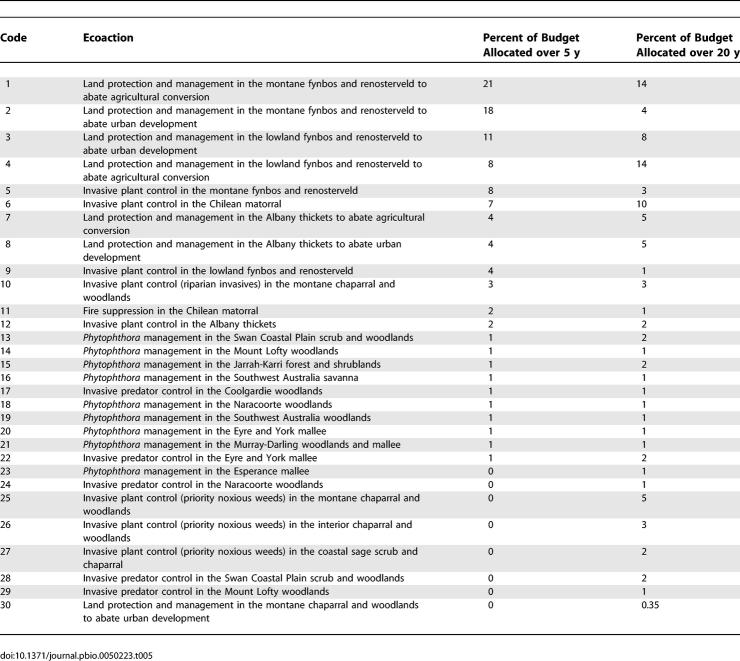
The Percent of the Total Budget (US$100 Million) Allocated to the 30 Ecoactions That Receive Investment over 20 y

Beyond the first 5 y, we see additional ecoactions prioritised for investment because further investments in initially selected ecoactions exhibit diminishing returns. Consequently, as one moves from a 5-y to a 20-y timeframe, the number of ecoactions identified for investment increases from 24 to 30, despite a 4-fold increase in the funds available. The greatest investment over 20 y is directed, in equal proportions to the montane and lowland fynbos and renosterveld ecoregions of South Africa, to the conservation action of land protection and management to abate agricultural conversion (both ecoregions are allocated approximately 14% of the total budget for investment in this conservation action; Table 5). Over 20 y all 17 ecoregions are allocated some funds (Table 5).

Overall, the investment schedule was insensitive to the annual available budget, though some of the lower-priority ecoactions did not receive funding when the annual budget was reduced to US$10 million. For example, under a reduced budget, the Coolgardie woodlands was the only ecoregion in Australia allocated investment in invasive predator control over 20 y, since the current level of investment in this ecoaction is small. Likewise, under a reduced budget, funding was not allocated to invasive plant control in the Californian/Baja Californian ecoregions.

### Comparison with Alternative Approaches

**Figure 4:**
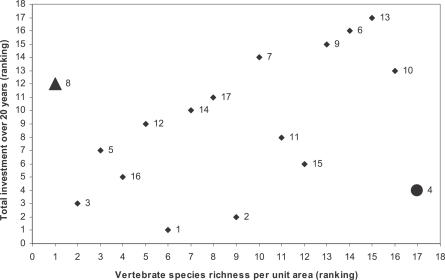
Scatter Plot of the Ecoregion Priorities When Ranked According to Total Investment and Vertebrate Species Richness Ecoregion codes are those provided in Figure 1, and vertebrate richness is represented by the richness of vertebrates per unit area (based on the total area of each ecoregion). Discrepancy between rankings is marked. The Chilean matorral ecoregion (denoted by a circle) has low species richness per unit area, but received a relatively large funding allocation over 20 y. The Jarrah-Karri forest and shrublands ecoregion (denoted by a triangle) has the greatest vertebrate species richness per unit area, but received a relatively small funding allocation over 20 y.

It is informative to compare the outcome of this analysis to that of a simpler analysis that ignores costs and benefits, and instead prioritises the ecoregions for investment on the basis of a single ecological criterion—in this case, vertebrate species richness per unit area. There was a lack of concordance (*r*_s_ = 0.39, *p* = 0.12) between the priorities based on the two approaches, indicating that they would recommend profoundly different ecoregions as investment priorities (Figure 4). For example, the Chilean matorral ecoregion has the fewest vertebrate species per unit area but received the fourth greatest allocation under the ecoaction approach because of the potentially high biodiversity benefit per dollar invested. Conversely, the Jarrah-Karri forest and shrublands ecoregion in Australia has the greatest vertebrate species richness per unit area but was not a priority using the ecoaction approach (Figure 4).

When we compared the ecoaction-specific framework to a simplified model of conservation that focuses only on land acquisition (see Materials and Methods), we found that greater biodiversity benefits are accrued by investing in actions targeted towards specific threats. The decision steps in the resource allocation process are identical regardless of the investment approach (Figure 2), with the exception that the land-acquisition-only approach considers only a single conservation action—land acquisition. Based on the data available for this analysis, we estimate that over 5 y many more species could be protected using an ecoaction approach (2,780 versus 703 species). After 20 y slightly more than twice as many species could be protected. The difference is reduced through time because of diminishing returns regardless of the investment approach. Therefore, after accounting for the existing level of investment in each ecoaction (which in some cases includes land acquisition with the costs of management added; Text S1) or in land acquisition alone (where the costs of managing specific threats are not accounted for; Table S1), greater returns can be achieved using the ecoaction-specific framework. These results were relatively insensitive to the parameterisation of the species–investment relationship, specifically, the rate at which the returns from investment diminish (as determined by the *z* exponent). The average ratio of species protected using the ecoaction approach and the land-acquisition-only approach over 5 y was approximately 3.49 (this ratio varied from 3.46 to 3.51 with the value of *z* for each ecoaction randomly chosen from a uniform distribution between 0.1 and 0.4, *n =* 30; see Materials and Methods).

## Discussion

These results illustrate the advantages of an ecoaction-specific framework over priority-setting approaches that ignore economic costs, or that focus only on the acquisition of land for protected areas. The Mediterranean example shows that an ecoaction-specific framework provides better outcomes for biodiversity conservation than the simpler approaches that have dominated the scientific literature [22]. In practice, very few conservation practitioners adopt species richness priorities identified by simple numerical ranking. Instead, they routinely consider the costs of investments, and more complex measures of biodiversity benefits. Our framework provides a standard, transparent, and quantitative template in which to solve complex resource allocation problems.

By specifying costs and benefits and a total budget, we produced an investment schedule that reveals shifting priorities through time as the returns from investment change. Because conservation budgets are often reallocated every year, it could be practical to follow flexible and time-varying investment schedules, as opposed to being tied to specific actions simply because they were previously regarded a high priority. Furthermore, if an equitable distribution of a base level of funds is important, then a pre-specified amount of funds could be allocated to each ecoregion, with funds directed to particular actions according to their relative return on investment.

Various refinements to our approach would be valuable. The calculation of biodiversity benefits could be improved by incorporating more detailed information from conservation practitioners, either in the form of empirical or expert data. We could also extend our analysis to consider other types of benefits, including the potential returns from the protection of ecosystem services [23]. Under such circumstances, it would be possible to assess the potential collateral benefits of conservation investments beyond the protection of biodiversity and to evaluate the trade-offs involved, as it is likely that different areas will be prioritised to achieve the alternative objectives. Other improvements might entail identifying the individual species that are most at risk due to the different threats, the impact of investment in each ecoaction on the persistence of these species, the likelihood of success of each ecoaction, and the potential for leverage.

As with typical conservation planning exercises that focus on protected area establishment, we have assumed that each ecoaction will be totally effective in abating the relevant threat. A plethora of factors (ranging from natural community succession to climate change) render this assumption unreliable [24,25]. It would be ideal if we had estimates of the likelihood of long-term success of each conservation action in conserving biodiversity, both for the duration of the action, and after investment ceases. These data are unlikely to be available at any time in the near future. Instead, we base the cost of each ecoaction on the assumption that enough funds are invested to have a high likelihood of success. Presently we assume that alleviating the most important threat will protect the species at risk, but the number of protected species will likely be overestimated if some species need to be protected from multiple threats that require different ecoactions. With knowledge of the individual species at risk due to each threat we could identify which species are affected by more than one threat. With this knowledge, the complementarity of each ecoaction in improving species persistence could be incorporated, and this would help to minimise the degree to which benefits are overestimated, assuming of course that all important threats have been identified. In addition, the assumption of diminishing returns with cumulative investment could, in some instances, be replaced with threshold relationships for those conservation actions that yield no benefit until some minimum level of investment is reached.

These, however, are straightforward technical modifications of the approach; obtaining the relevant data represents the greatest challenge. Within our dynamic framework, the investment schedules can be updated as our knowledge improves. Application of our framework can also provide insights into research priorities. For example, through our Mediterranean application it has become apparent that information on the likelihood of success and patterns in threat co-variation among species are important subjects of future research. We hope that our framework for conservation investment will encourage conservation practitioners to track and report action-specific data to allow a refined framework to be parameterised.

To examine the sensitivity of our results to the budget, we reduced the amount of money available per annum from US$100 million to US$10 million. In this example, varying the annual budget simply altered what was able to be achieved over the timeframe of interest: an investment of US$10 million over 10 y will achieve approximately the same outcomes as an investment of US$100 million in 1 y. This is because the investment schedules are determined only by the area requiring investment and the relative returns of the investment. While the “maximise short-term gain” heuristic closely approximates the optimal solution, especially with funding uncertainty, the urgency of investment could also be incorporated if information were available on the rate of species loss in each ecoregion due to each threat [4]. Under such circumstances, the investment schedules would change over different timeframes because of the rates of species loss influencing both the area requiring each ecoaction and the relative return from investment. Explicitly accounting for ongoing species losses would change our objective to “minimising losses” rather than “maximising gains” [4,6,26]. Incorporating information on the rates of species loss would further improve the ability to determine when conservation actions should be implemented in order to achieve the greatest outcomes for biodiversity. Presently, data on the rates of loss of species due to particular threats are scarce, and there is limited understanding of how species loss varies with changes in available habitat.

We have applied the approach at a global scale, but it will be more effectively applied at local or regional scales, if only because in many cases the required data are more likely to be available and to be more accurately estimated. Application at a global scale is nevertheless important, despite the sparseness of the data. First, global non-governmental organisations and international agencies are interested in decision-making at a global scale, and will make investment decisions at such a scale. Second, there is now a large academic literature on setting conservation priorities at a global scale; these studies are equally beset by sparse data and poorly tested assumptions, they have mainly ignored costs, and they have focussed on protected area establishment.

Analysis at a finer spatial scale would further increase the efficiency of the investment schedules by accounting for the heterogeneity in the costs and benefits of conservation actions. Such an analysis will likely require assessment of empirical, modelled, and expert data. With a more detailed and refined analysis we could also account for the actual costs and relative success of conservation actions undertaken in the past. Such an analysis could also allow finer-scale socio-economic and policy data to be incorporated. For example, the area of land predicted to be vulnerable to agricultural conversion in the montane fynbos and renosterveld ecoregion of South Africa is likely to be overestimated by the biophysical models employed as these ignore socio-economic and political factors. The collapse of subsidies in this region may mean that only small areas are currently experiencing conversion pressures [19]. While analysis at a finer scale would allow a refined assessment of investment priorities, it would be at the expense of the global-scale evaluation of investment priorities presented here. By being transferable across scales, our framework can help to bridge the current gap between global-scale analyses and investment decisions that are implemented within regions, as it can provide an understanding of the relative importance of each ecoaction for conserving biodiversity within a global context.

Regardless of scale, stakeholders and experts are integral to the success of the ecoaction approach, through identifying threats and actions, determining the relative costs and benefits of each action, and identifying local constraints for their implementation (see Materials and Methods). The results of any assessment must also be interpreted in the context of the value systems of stakeholders, as well as other factors such as the implementation capacity of the relevant management agencies. These factors reflect the fine-tuning of quantitative analyses that is required to account for real-world constraints and opportunities, although the aim is to avoid such post hoc refinements and integrate all important considerations into the analysis. The results of a systematic and transparent assessment make explicit any trade-offs, compromises, and opportunities. When we qualitatively compare the results of our analyses to planning approaches within South Africa [27], we find a high degree of concordance, as we do when we compare the relative levels of investment in *Phytophthora* management and predator control in Australia [28–30]. Nevertheless, the identification of investment priorities through a systematic and transparent process will be extremely useful when local experts are not available or there is a need to remove individual biases. At a global or even national scale, there is likely to be a deficit of experts with knowledge of multiple regions and conservation actions and an ability to identify investment schedules across these in an integrated manner.

Our conservation investment framework offers substantial dividends for biodiversity conservation by prioritising the most appropriate and feasible conservation actions to abate the threats that operate in a region. Already, there has been a call for conservation organisations to audit their investments and measure their returns [31–34]. For conservation practitioners, this framework represents a much needed tool for incorporating their insights and experience regarding the costs, benefits, and dynamics of a suite of conservation actions to maximise conservation outcomes.

## Supporting Information

Table S1 Data for Funding Allocation Analysis Involving Only Land Acquisition (50 KB DOC)

Text S1 Data Obtained for Each Ecoregion to Apply Our Ecoaction-Specific Framework (234 KB DOC)

Text S2 Derivation of the Parameter α (25 KB DOC)

## Abbreviations

IUCN: World Conservation Union
