# Supplementary material for: Conserving Biodiversity Efficiently: What to Do, Where, and When
Source: PLoS Biol. 2007 Aug 21;5(9):e223. doi: 10.1371/journal.pbio.0050223 (PMC1950771; doi:10.1371/journal.pbio.0050223)
Supplement: Supplementary file 1 [file pbio.0050223.sd001.doc]

**Text S1. Data obtained for each ecoregion to apply**

**our ecoaction-specific framework**

This supporting information outlines the data obtained to apply our ecoaction-specific framework to the mediterranean ecoregions of Australia, Chile, South Africa, California and Baja California. For each of the priority conservation actions identified to abate the key threats in each ecoregion, we determined: (1) the cost per unit area, (2) the area already receiving the action, and (3) the area requiring the action.

**Australian mediterranean ecoregions**

*Introduced predators (specifically, cats (*Felis catus*) and foxes (*Vulpes vulpes*))*

We assume that invasive predator control could be applied to the entire vegetated area within the Australian mediterranean ecoregions, after accounting for the area already receiving invasive predator control [1]. Fox and cat baiting (using “1080” baits and accounting for labour) costs approximately US$66 and US$289 per km^2^ respectively [1-3]. Native vertebrates in Western Australia have a high tolerance to “1080” due to the co-evolution with native peas (*Gastrolobium* spp.), which has the same active ingredient as is in “1080” (sodium monofluoroacetate) [1,2]. However, the same tolerance does not exist in the eastern Australian ecoregions and the search for a canid- (and also felid-) specific control technique continues. We therefore add to the cost of baiting the cost of fox and cat control research across all ecoregions; US$2.92 million and US$0.73 million per annum respectively [4]. Overall, we estimate the cost of invasive predator control (materials, labour, and research) to be US$355 per km^2^ and since this is an annual cost, we endow this value over 20 years to obtain US$5,237 per km^2^. We add to this the costs associated with establishing conservation covenants (flat rate of US$2,555 per km^2^) on the unprotected (not within IUCN I-IV protected areas) proportion of land requiring this action, as this action is likely to require a formal arrangement with landholders in order to proceed on private land. Although there is evidence that invasive predator control might benefit native plants [5] (for example, through reducing the effects of competitive exclusion) the action is largely vertebrate-specific and therefore we base the calculation of biodiversity benefits solely on vertebrates (Table 1).

Table 1. Data employed for the conservation action of invasive predator control in the ten Australian mediterranean ecoregions.

| **Ecoregion** | **Coolgardie woodlands** | **Esperance mallee** | **Eyre and York mallee** | **Jarrah-Karri forest and shrublands** | **Swan Coastal Plain scrub and woodlands** | **Mount Lofty woodlands** | **Murray-Darling woodlands and mallee** | **Naracoorte woodlands** | **Southwest Australia savanna** | **Southwest Australia woodlands** |
| --- | --- | --- | --- | --- | --- | --- | --- | --- | --- | --- |
| **Biodiversity benefit** | 137 | 153 | 153 | 115 | 143 | 157 | 200 | 150 | 183 | 135 |
| **Cost (US$/km^2^)** | 7,540 | 6,923 | 7,091 | 6,914 | 7,125 | 6,942 | 7,259 | 6,981 | 7,548 | 7,559 |
| **Total area (km^2^)** | 137,226 | 115,460 | 60,896 | 10,455 | 15,210 | 23,786 | 197,895 | 27,531 | 168,713 | 46,015 |
| **Area requiring action (km^2^)** | 136,029 | 48,709 | 18,510 | 343 | 4,606 | 3,532 | 116,056 | 3,909 | 65,596 | 7,026 |
| **Area already receiving action (km^2^)** | 0 | 17,633 | 416 | 7,532 | 1,226 | 1,221 | 5,973 | 986 | 6,150 | 16,567 |

*Soil-borne pseudo-fungus,* Phytophthora cinnamomi

For nine ecoregions, we calculate the area requiring management to abate *Phytophthora cinnamomi* spread as the remaining susceptible vegetated area within 85 kilometres of the coast, after accounting for the area already receiving management. The Coolgardie woodlands ecoregion is considered to not be susceptible to *Phytophthora*. For three ecoregions (Jarri-Karri forest and shrublands, Swan Coastal Plain shrub and woodlands, and Southwest Australia woodlands), we do not restrict the susceptible vegetated area to within 85 kilometres of the coast, as these ecoregions are considered sufficiently moist for *Phytophthora* infestations to occur throughout [6-8]. In the Esperance mallee ecoregion, five km^2^ has been treated with phosphite at a cost of US$34,748 per km^2^, which includes the cost of phosphite application, monitoring and planning, and mapping. In other ecoregions there has been very limited treatment, although extensive quarantine operations have been undertaken [8]. Pathogen resistance lasts for approximately one year [9], and therefore we endow the cost of phosphite application over 20 years. We add to this the costs associated with risk mapping, policy development, communication, and research (US$18.65 per km^2^) [10]. We endow the cost of research over five years. We add to the overall cost that associated with establishing conservation covenants (flat rate of US$2,555 per km^2^) on the unprotected (not within IUCN I-IV protected areas) proportion of land requiring this action, as this action is likely to require a formal arrangement with landholders in order to proceed on private land (Table 2).

Table 2. Data employed for the conservation action of *Phytophthora* *cinnamomi* management in nine of the Australian mediterranean ecoregions.

| **Ecoregion** | **Esperance mallee** | **Eyre and York mallee** | **Jarrah-Karri forest and shrublands** | **Swan Coastal Plain scrub and woodlands** | **Mount Lofty woodlands** | **Murray-Darling woodlands and mallee** | **Naracoorte woodlands** | **Southwest Australia savanna** | **Southwest Australia woodlands** |
| --- | --- | --- | --- | --- | --- | --- | --- | --- | --- |
| **Biodiversity benefit** | 294 | 251 | 222 | 256 | 255 | 254 | 223 | 276 | 233 |
| **Cost (US$/km^2^)** | 514,424 | 514,592 | 514,415 | 514,626 | 514,443 | 514,760 | 514,482 | 515,049 | 515,060 |
| **Total area (km^2^)** | 115,460 | 60,896 | 10,455 | 15,210 | 23,786 | 197,895 | 27,531 | 168,713 | 46,015 |
| **Area requiring action (km^2^)** | 27,453 | 12,791 | 5,364 | 4,985 | 4,198 | 901 | 3,097 | 14,140 | 22,960 |
| **Area already receiving action (km^2^)** | 5 | 0 | 0 | 0 | 0 | 0 | 0 | 0 | 0 |

*Habitat fragmentation*

We identify five ecoregions with less than 50 percent of their original native vegetation cover (National Vegetation Information System, stage 1, version 3.1) (Table 3). We prioritise 15 percent of the unvegetated area of each ecoregion for revegetation and we consider the area to already receive this action as that which is currently vegetated (Table 3). We derive revegetation costs from a survey of revegetation activities in Australia [11] (flat rate of US$299,300 per km^2^). This action is likely to require a formal arrangement with landholders in order to ensure it is long-lasting and transferred to all successive landholders. We therefore add to the cost of revegetation, the cost of establishing conservation covenants (flat rate of US$2,555 per km^2^) on the unprotected (not within IUCN I-IV protected areas) proportion of the land requiring this action (Table 3).

Table 3. Data employed for the conservation action of revegetation in five of the Australian mediterranean ecoregions.

| **Ecoregion** | **Eyre and York mallee** | **Swan Coastal Plain scrub and woodlands** | **Mount Lofty woodlands** | **Naracoorte woodlands** | **Southwest Australia savanna** |
| --- | --- | --- | --- | --- | --- |
| **Biodiversity benefit** | 514 | 565 | 517 | 414 | 519 |
| **Cost (US$/km^2^)** | 301,154 | 301,188 | 301,005 | 301,044 | 301,611 |
| **Total area (km^2^)** | 60,896 | 15,210 | 23,786 | 27,531 | 168,713 |
| **Area requiring action (km^2^)** | 6,295 | 1,407 | 2,855 | 3,395 | 14,545 |
| **Area already receiving action (km^2^)** | 18,926 | 5,831 | 4,753 | 4,895 | 71,746 |

**Chilean mediterranean ecoregion**

*Invasive plants*

Presently, there is no formal invasive plant control program in Chile and detailed mapping of invaded areas is not available. We identify natural and semi-natural habitat vulnerable to non-native plant invasion using a spatially-explicit, rule-based model, based on distance to roads and elevation; two factors considered important determinants of invasive plant occurrence in Chile [12,13]. Areas within 200 metres of roads are considered more vulnerable to invasion by non-native plants, and we allocate the first 100 metres twice the weight as the second 100 meters [14]. In Chile, many invasive plants are not as prevalent in areas of high elevation, likely as a consequence of their Eurasian origin [12,13]. We give a higher weight to elevation than distance to roads since if an area is unsuitable on the basis of elevation it is likely to be unsuitable even if a road was present. We generate an index value for elevation ranging from zero to 20. We assign areas at zero meters elevation an index value of 20, and areas over 3,500 meters an index value of zero. Without information to the contrary, we assume the presence of roads and elevation are independent and derive the overall rankings by taking the sum. We predict that areas of natural and semi-natural habitat in the highest quartile of invasion rankings to be currently invaded by non-native plants and to require invasive plant control through removal of plants, herbicide application, and revegetation (Table 4).

Due to a lack of invasive plant control data for Chile, we use average costs associated with the removal of young plants and seedlings of *Acacia* spp. across all density classes from South Africa (see South Africa ecoregions section). We adjust these costs for the purchasing power of the Chilean Peso relative to the purchasing power of the South African Rand. We estimate the costs associated with revegetation of previously invaded areas using the costs associated with planting, training, establishing nurseries, fencing, and public education, which equates to US$126,757 per km^2^ (Text S1). This cost includes educational activities for local people and the costs associated with fencing, likely for control of livestock and rabbits [12,15] (Table 4).

*Altered fire regimes*

To determine the area requiring fire suppression, we assess the location of fires within the mediterranean ecoregion since 2000 and assume that the associated land cover classes might be susceptible to fires in the future [16,17]. The total government budget for fire suppression in Chile is US$10,890,922, which when added to the costs of fire mitigation (US$1,013,563) amounts to US$11,904,485. This budget is for the area considered by CONAF to be at risk of fire across all of Chile (337,195 km^2^). The total cost is therefore US$35 per km^2^, which we endow over 20 years. Within the area considered at risk of fire, 61,032 km^2^ falls within the mediterranean ecoregion and currently receives fire suppression (Table 4).

*Conversion of natural habitat*

We determine the area requiring land acquisition for reserve establishment using priority area maps for reserve establishment from the Chilean National Biodiversity Strategy [18] We consider IUCN I-IV protected areas to already receive this action. We estimate the average cost of land to be US$150,000 per km^2^, based on the selling prices of farms in the mediterranean ecoregion ([http://www.farmschile.com](http://www.farmschile.com/)). Since most newly acquired land in central Chile will require extensive restoration to allow biodiversity to recover, we add to the acquisition cost that associated with fire suppression (which we endow over 20 years) and invasive plant control (Table 4).

Table 4. Data employed for the conservation actions of invasive plant control, fire suppression, and land acquisition in the Chilean mediterranean ecoregion.

|  | **Invasive plant control** | **Fire suppression** | **Land acquisition** |
| --- | --- | --- | --- |
| **Biodiversity benefit** | 1,337 | 499 | 2,089 |
| **Cost (US$/km^2^)** | 126,757 | 516 | 277,273 |
| **Total area (km^2^)** | 148,383 | 148,383 | 148,383 |
| **Area requiring action (km^2^)** | 7,080 | 23,159 | 14,640 |
| **Area already receiving action (km^2^)** | 0 | 61,032 | 1,332 |

**South African mediterranean ecoregions**

*Conversion of natural habitat (due to agriculture expansion and urban development)*

To address agriculture expansion and urban development we identify a conservation action that comprises a combination of land acquisition, off reserve management, and ongoing management (herein terms land protection and management). We estimate the area requiring land protection and management to abate these two threats as the land highly suitable for dryland cultivation or urban development based on existing land use models [19-21] and that is also unconverted [22] and unprotected [23]. Within the area requiring land protection and management, we consider the areas to already receive this action to be Type 1 statutory protected areas: National Parks, Provincial Nature Reserves, Local Authority Nature Reserves, and Department of Water Affairs and Forestry Nature Reserves [23]. We determine the cost associated with this action for each ecoregion (see Equation 1 and Table 5).

$(\text{Cost}(1)\times\varepsilon)+(\text{Cost}(2)\times\delta)+(\sigma)$	Equation 1

*Invasive plants*

We estimate the area requiring invasive plant control to be the unconverted area that is currently densely invaded [21,22]. We obtain the costs associated with clearing areas densely invaded with *Acacia* species and the area of dense stands (75-100 percent density class) that already receives invasive plant control (across both the Western and Eastern Cape Provinces) from data collated by the Working for Water program. Overall, 19.3 km^2^ of densely invaded stands of *Acacia* (of 405 km^2^ of invaded area across all density classes) have been cleared in the Eastern and Western Cape Provinces and we assume that these stands occur entirely within our three ecoregions of interest. The costs associated with clearing dense stands of invasive plants account for the stature of the plants (mature, young, or seedling), the initial clearing costs plus three follow-up treatments, and the cost of herbicides. Additional costs that we do not account for include those of reintroducing species, soil stabilisation on slopes, and fire management. We recognise that invasive plant control can also reduce the spread of invasive plants to presently unaffected areas and therefore the benefit of this action will extend beyond the area receiving the action, but we do not account for this effect in our calculations due to a lack of data (Table 5).

Table 5. Summary of variables used to determine the costs associated with abating agricultural conversion and urban development.

| Variable | Description | Source/Method of calculation | Value (US$ per km^2^) |
| --- | --- | --- | --- |
| $\text{Cost}(1)$ | Ecoregion-specific cost of acquiring unimproved land | Land acquisition costs + Invasive plant control costs + Operating costs [24].  We adjust land acquisition costs from 2000 for inflation and changes in exchange rates.  Invasive plant control costs reflect the average costs associated with clearing invasive plants of *Acacia* spp. across all density classes and for the purchase of herbicides.    Operating costs account for overheads, staffing, and ongoing management including invasive plant control and we estimate these from 2000 values adjusted for inflation and changes in exchange rates. | Land acquisition costs  Albany Thickets $48,100  Lowland Fynbos $46,500  Montane Fynbos $10,400  Invasive plant control costs  $14,600  Operating costs  $18,400 (endowed over 20 years)  Summary Cost(1)  Albany Thickets $81,100  Lowland Fynbos $79,500  Montane Fynbos $43,400 |
| $\varepsilon$ | Ecoregion-specific proportion of effort allocated to the action of land acquisition | Reflects opportunities for land acquisition in each ecoregion within a five kilometre buffer of existing protected areas [24] with the aim to protect the areas at risk of habitat conversion, with adjustment of the mechanisms depending on the constraints and opportunities for alternative forms of protection. | Albany Thickets 0.74  Lowland Fynbos 0.3  Montane Fynbos 0.58 |
| $\text{Cost}(2)$ | Cost of off-reserve management | Invasive plant control costs + Operating costs + Legal costs [24].  We assume invasive plant control costs to be 80 percent of the full costs.  Operating costs account for monitoring and extension services. We estimate these costs from 2000 values adjusted for inflation and changes in exchange rates. | Invasive plant control costs  $11,700  Operating costs  $14,800 (endowed over 20 years)  Legal costs  $100  Summary Cost(2)  $26,600 |
| $\delta$ | Ecoregion-specific proportion of effort allocated to the action of off-reserve management. | Reflects opportunities for off-reserve management in each ecoregion [24] | Albany Thickets 0.26  Lowland Fynbos 0.7  Montane Fynbos 0.42 |
| $\sigma$ | Costs of scientific services, and head and regional offices costs | We calculate these costs as a fixed proportion of annual operating costs [24]  Scientific services = 10 percent per annum  Head office = 7 percent per annum  Regional office = 8 percent per annum. | Scientific services = $125 per annum  Head office = $88 per annum  Regional office = $100 per annum  Summary  $4,400 (endowed over 20 years) |

Table 6. Data employed for the conservation action of land protection and management to abate agricultural conversion in the three South African mediterranean ecoregions.

| **Ecoregion** | **Albany thickets** | **Lowland fynbos and renosterveld** | **Montane fynbos and renosterveld** |
| --- | --- | --- | --- |
| **Biodiversity benefit** | 506 | 1,043 | 2,005 |
| **Cost (US$/km^2^)** | 71,330 | 46,870 | 40,744 |
| **Total area (km^2^)** | 17,135 | 32,764 | 45,746 |
| **Area requiring action (km^2^)** | 1,380 | 8,232 | 6,761 |
| **Area already receiving action (km^2^)** | 31 | 215 | 185 |

Table 7. Data employed for the conservation action of land protection and management to abate urban development in the three South African mediterranean ecoregions.

| **Ecoregion** | **Albany thickets** | **Lowland fynbos and renosterveld** | **Montane fynbos and renosterveld** |
| --- | --- | --- | --- |
| **Biodiversity benefit** | 570 | 1,246 | 2,466 |
| **Cost (US$/km^2^)** | 71,330 | 46,870 | 40,744 |
| **Total area (km^2^)** | 17,135 | 32,764 | 45,746 |
| **Area requiring action (km^2^)** | 1,368 | 3,496 | 2,177 |
| **Area already receiving action (km^2^)** | 87 | 201 | 163 |

Table 8. Data employed for the conservation action of invasive plant control in the three South African mediterranean ecoregions.

| **Ecoregion** | **Albany thickets** | **Lowland fynbos and renosterveld** | **Montane fynbos and renosterveld** |
| --- | --- | --- | --- |
| **Biodiversity benefit** | 267 | 545 | 1,043 |
| **Cost (US$/km^2^)** | 92,900 | 92,900 | 92,900 |
| **Total area (km^2^)** | 17,135 | 32,764 | 45,746 |
| **Area requiring action (km^2^)** | 544 | 299 | 605 |
| **Area already receiving action (km^2^)** | 3 | 7 | 9 |

**Californian ecoregions**

*Conversion of natural habitat (due to urban development)*

We identify the area requiring protection to abate urban development as natural and semi-natural habitat within the extent of the projected urban growth footprint for 2020 [25]. We base estimates for the Mexico portion of the Coastal Sage shrub and chaparral ecoregion on the proportion of natural and semi-natural habitat predicted to experience urban development in the Californian extent of this ecoregion (equating to 5.29 percent). We subtract from the area requiring protection the area already protected. In California this is considered to be the GAP status 1-3 protected areas, TNC fee acquired areas and easements, and areas within the General Plan ‘Open Space’ category. We identify the Fundación la Puerta private reserve to be in the vicinity of potential urban growth in Mexico and we assume that the region near Sierra San Pedro Mártir National Park (at high elevations) and Isla Gaudalupe (an island with small fishing towns) will not experience significant urban growth (Table 9).

We identify three conservation actions to abate urban development: land acquisition, conservation easements, and local land use planning activities. We base the costs of land acquisition and easements on the average values of transactions conducted within the Californian extent of the three mediterranean ecoregions by The Nature Conservancy of California from 2002-2005. There were 18 land purchases in this period with an average value of US$1,968,194 per km^2^ and eight easement purchases with an average value of US$1,102,831 per km^2^. General Plans, or land use zoning plans, form the third conservation action. These plans, required by California state law, represent the city and county’s blueprint for future development [26]. We base our cost data for this action on the Monterey County 2006 General Plan Update, estimated to have cost US$91 per km^2^ (we assume that the costs for different counties will not vary and endow this value over 20 years). We also assume that land acquisition, conservation easements, and local land use planning activities could occur in equal proportions throughout the ecoregions and we therefore weight them equally to obtain US$1,013,468 per km^2^ (Table 9) [27,28].

There is an additional conservation planning activity of particular relevance in some regions of California: the Natural Community Conservation Planning (NCCP) program. This activity identifies areas that are important for conservation of native diversity, including species listed under the Endangered Species Act. A suite of regulatory, market, and funding mechanisms are then established to facilitate the implementation of the conservation plan. We did not include this action, however, because of the complexity of estimating its costs.

Table 9. Data employed for the conservation action of land protection to abate urban development in the three Californian mediterranean ecoregions.

| **Ecoregion** | **Coastal Sage scrub and chaparral** | **Interior chaparral and woodlands** | **Montane chaparral and woodlands** |
| --- | --- | --- | --- |
| **Biodiversity benefit** | 315 | 359 | 350 |
| **Cost (US$/km^2^)** | 1,013,882 | 1,013,882 | 1,013,882 |
| **Total area (km^2^)** | 36,249 | 64,627 | 20,407 |
| **Area requiring action (km^2^)** | 1,273 | 307 | 104 |
| **Area already receiving action (km^2^)** | 977 | 86 | 30 |

*Altered fire regime*

We consider the area requiring fire management to be the protected areas and public lands within California (GAP status 1-3 protected areas in California and TNC fee acquired areas). We omit conservation easements since there is no guarantee that fire management can be conducted on private land. We do not include protected areas in the Mexico portion of the Coastal Sage shrub and chaparral ecoregion as Sierra San Pedro Mártir National Park experiences a natural fire regime [29] and studies indicate that the absence of fire suppression in California Baja has resulted in a diverse patch mosaic of chaparral stands which experiences low-intensity fires [30,31]. In contrast, fire management in southern California, has resulted in fewer but much larger fire events [30]. We subtract, from the area requiring fire management, the protected area with fire regimes within or near historical ranges (as mapped in the Fire Regime Condition Class by the California Department of Forestry and Fire Protection) [32] (Table 10).

We consider two fire management actions and cost these actions using information acquired from the US Forest Service Region 5. For the Coastal Sage scrub and chaparral ecoregion we consider fire suppression to abate high frequency, anthropogenic-caused fires. We estimate the cost of this action using data from the Cleveland and the San Bernardino National Forests (estimates for FY 2006 provided by the US Forest Service Region 5) that are located within the ecoregion. We use the median value of the costs of two burn sizes (0.25-100 acres and 10-100 acres) which is US$110,061 per km^2^ per annum for the suppression of fire (we assume that suppression is required every five years and that the timing does not vary with vegetation type). For the Coastal Sage scrub and chaparral ecoregion, we also include the costs of ecological studies on the effects of fire suppression. Based on estimates from The Nature Conservancy’s management of the Irvine Ranch Land Reserve in southern California, we estimate this to be an annual cost of US$657 per km^2^. We endow the combined costs over 20 years (Table 10).

For the Interior chaparral and woodland and Montane chaparral and woodland ecoregions we consider fuel reduction to reduce the likelihood of catastrophic fires. We use data from the US Forest Service Region 5 on fuel reduction treatment for three National Forests that occur within these two ecoregions: the Angeles, Los Padres, and San Bernadino forests. The average annual cost for mechanical and prescribed fuel reduction within these forests during 2002-2005 was US$35,707 per km^2^ (costs are inclusive of overhead, personnel, report documents, data entry etc) and we endow this cost over 20 years. We assume this cost is incurred intermittently (every five years) and that the costs and timing does not vary with vegetation type (Table 10).

Table 10. Data employed for the conservation action of fire management in the three Californian mediterranean ecoregions.

| **Ecoregion** | **Coastal Sage scrub and chaparral** | **Interior chaparral and woodlands** | **Montane chaparral and woodlands** |
| --- | --- | --- | --- |
| **Biodiversity benefit** | 247 | 374 | 368 |
| **Cost (US$/km^2^)** | 1,633,358 | 526,765 | 526,765 |
| **Total area (km^2^)** | 24,337 | 64,627 | 20,407 |
| **Area requiring action (km^2^)** | 5,574 | 13,193 | 13,228 |
| **Area already receiving action (km^2^)** | 1,988 | 6,346 | 2,280 |

*Invasive plants*

We use two approaches to address the threat of invasive plants and, in the absence of consistent state-wide or county-level databases, base these estimates on two case study areas.

***(i) Control of 'priority noxious weeds’ on public lands***
We estimate the area requiring invasive plant control on public lands as 0.51 percent of public lands and protected areas (GAP status 1-3 protected areas in California, TNC fee acquired areas, and three protected areas in Mexico - Isla Guadalupe, Sierra San Pedro Mártir, and Fundación la Puerta). We generate this proportion based on data provided by the National Park Service (NPS) for Santa Monica Mountains National Recreation Area (SMMNRA) for 2006. The NPS is responsible for approximately 89 km^2^ of the SMMNRA of which 0.46 km^2^ (0.51 percent; estimated from surveys) is predicted to be invaded by 19 priority invasive species that are actively controlled. Although this proportion is relatively low, three points must be noted; (a) This figure reflects the amount of land invaded by noxious weeds of high priority for control, and it does not reflect areas that are considered too invaded to justify treatment (e.g., low lying non-native grasslands); (b) the data are from primarily chaparral areas which are generally considered to be more resistant than other communities; and (c) the NPS generally acquires and manages high quality habitat. We subtract, from this area, the area already receiving invasive plant control; 26.19 percent of the area based on that treated by the NPS at SMMNRA (0.12 km^2^ of the 0.46 km^2^ invaded) (Table 11).

The costs of invasive plant control of priority noxious species by the NPS within the SMMNRA area equates to US$375,000 over 0.12km^2^ for two years of control (using herbicide, by mechanical means, and via hand pulling techniques), revegetation (via seed collection, plant propagation, and planting), and post-revegetation monitoring and experimental research. These costs include the hire of temporary employees and one-sixth of a permanent full-time employee for managing control activities ($10,000 per year for each of the two years). We therefore estimate the total cost of controlling priority noxious weeds on public land to be US$3,300,000 per km^2^ (Table 11).

Table 11. Data employed for the conservation action of invasive plant control on public lands in the three Californian mediterranean ecoregions.

| **Ecoregion** | **Coastal Sage scrub and chaparral** | **Interior chaparral and woodlands** | **Montane chaparral and woodlands** |
| --- | --- | --- | --- |
| **Biodiversity benefit** | 839 | 1497 | 1470 |
| **Cost (US$/km^2^)** | 3,300,000 | 3,300,000 | 3,300,000 |
| **Total area (km^2^)** | 36,249 | 64,627 | 20,407 |
| **Area requiring action (km^2^)** | 31 | 67 | 67 |
| **Area already receiving action (km^2^)** | 8 | 18 | 18 |

***(ii) Control of riparian invasives through watershed-based Weed Management Areas***

To include watershed-scale control activities that occur on private lands we estimate the area requiring this action as 26 percent of the 100-year floodplain in California [33], which is below 350 metres elevation, is not open-standing water or estuaries, and not public or TNC fee acquired lands which might be accounted for in (*i*). In the absence of floodplain data for Mexico, we buffer major rivers (less than 350 metres elevation, excluding standing water or estuaries, and unprotected) by 250 metres – the average width of floodplains in California as determined by visual map inspection. We estimate the area invaded by *A. donax* (and *Tamarix* spp.) based on data compiled by the Santa Margarita and San Luis Rey Weed Management Areas ([www.smslrwma.org/invasives/Arundo/ADRegionalMap.html](http://www.smslrwma.org/invasives/Arundo/ADRegionalMap.html)). Of a total of 3,935,997 meters of rivers and streams surveyed, *A. donax* and/or *Tamarix* spp. was present along 1,008,318 meters (26 percent) and we extrapolate this value to the measured floodplain extent. We subtract, from the estimated area of private land requiring invasive plant control, the proportion of the invaded area already cleared of *A. donax* or where *A. donax* clearing is underway. This equates to 245,589 meters or 24 percent of the invaded area (Table 12).

We base the cost of riparian invasive species control on the San Luis Rey Weed Management Area. The cost of controlling *A. donax* and *Tamarix* spp. is US$4,447,897 per km^2^ over 10 years. This includes US$3,706,581 for five years of initial treatment (herbicide and mowing), followed by revegetation, and periodic treatment for the next five years. The cost also includes that associated with securing permits to work on private land. This estimate might be relatively low compared to other areas since this watershed has detailed maps of invasion density and experienced crews to undertake control activities (Table 12).

One consideration in including invasive plant control activities in riparian areas is that although these areas are of small spatial extent, similar to vernal pools and coastal dunes, they harbour a disproportionate amount of biodiversity [34]. Consequently, a species-area curve based on the entire ecoregion may not reflect the true gains of conservation activities in riparian areas.

Table 12. Data employed for the conservation action of invasive riparian plant control on private lands through watershed-based efforts in the three Californian mediterranean ecoregions.

| **Ecoregion** | **Coastal Sage scrub and chaparral** | **Interior chaparral and woodlands** | **Montane chaparral and woodlands** |
| --- | --- | --- | --- |
| **Biodiversity benefit** | 839 | 1497 | 1470 |
| **Cost (US$/km^2^)** | 4,447,897 | 4,447,897 | 4,447,897 |
| **Total area (km^2^)** | 36,249 | 64,627 | 20,407 |
| **Area requiring action (km^2^)** | 208 | 445 | 14 |
| **Area already receiving action (km^2^)** | 51 | 108 | 3 |

*Conversion of natural habitat (due to agriculture expansion)*

We estimate the area requiring protection from conversion to agriculture as the unconverted, pre-settlement extent of oak woodland, oak forest, and grassland in California and Mexico [35,36] that is below 800 meters elevation (identified as a threshold for vineyard cultivation [37]). For the Coastal Sage scrub and chaparral ecoregion we also include all areas of scrub at any elevation as vulnerable to agricultural conversion. We subtract, from the area requiring protection, the areas that are protected: GAP status 1-3 protected areas, TNC fee acquired areas and easements, the General Plan ‘Open Space’ category in California, and the three protected areas in Mexico (Isla Guadalupe, Sierra San Pedro Mártir, and Fundación la Puerta) (Table 13).

The conservation actions to abate this threat and the cost of those actions are the same as those for urban development: land acquisition, conservation easements, and local land use planning. These actions, however, do not include other conservation activities that target specific types of agriculture or activities that address areas of established agriculture, e.g., vineyard certification schemes or education and outreach.

Table 13. Data employed for the conservation action of land protection to abate agricultural expansion in the three Californian mediterranean ecoregions.

| **Ecoregion** | **Coastal Sage scrub and chaparral** | **Interior chaparral and woodlands** | **Montane chaparral and woodlands** |
| --- | --- | --- | --- |
| **Biodiversity benefit** | 770 | 986 | 966 |
| **Cost (US$/km^2^)** | 1,013,882 | 1,013,882 | 1,013,882 |
| **Total area (km^2^)** | 36,249 | 64,627 | 20,407 |
| **Area requiring action (km^2^)** | 12,021 | 39,596 | 3,735 |
| **Area already receiving action (km^2^)** | 5,362 | 10,231 | 2,183 |

**References**

1. Reddiex B, Forsyth DM, McDonald-Madden E, Einoder LD, Griffioen PA, et al. (2004) Review of existing red fox, wild dog, feral cat, rabbit, feral pig, and feral goat control in Australia. 1. Audit. Melbourne, Australia: Arthur Rylah Institute for Environmental Research, Department of Sustainability and Environment.

2. Wyre G (2004) Management of the Western Shield program: Western Shield review - February 2003. Conservation Science of Western Australia 5: 20-30.

3. Algar D, Burrows ND (2004) Feral cat control research: Western Shield review - February 2003. Conservation Science of Western Australia 5: 131-163.

4. Bomford M, Hart Q (2002) Non-indigenous vertebrates in Australia. In: Pimentel D, editor. Biological Invasions: Economic and Environmental Costs of Alien Plant, Animal and Microbe Species. Florida: CRC Press. pp. 25-45.

5. Shepherd KA, Wardell-Johnson GW, Loneragan WA, Bell DT (1997) Diet of herbivorous marsupials in a Eucalyptus marginata forest and their impact on the understorey vegetation. Australian Forestry 80: 47-54.

6. Wardell-Johnson G, Nichols O (1991) Forest wildlife and habitat management in southwestern Australia: knowledge, research and direction. In: Lunney D, editor. Conservation of Australia's Forest Fauna. Mosman: Royal Zoological Society of NSW. pp. 161-192.

7. Garkaklis MJ, Calver MC, Wilson BA, G.E. St. J. Hardy (2004) Habitat alteration caused by an introduced plant disease, Phytophthora cinnamomi, a potential threat to the conservation of Australia's forest fauna. In: Lunney D, editor. Conservation of Australia's Forest Fauna. Mosman: Royal Zoological Society of New South Wales. pp. 899-913.

8. Shearer B (1989) Dieback of native plant communities caused by Phytophthora species - a major factor affecting land use in South-Western Australia. Land and Water Research News 5: 15-26.

9. Barrett S. Monitoring of aerial phosphite applications for the control of *Phytophthora cinnamomi* in the Albany District. In: McComb JA, G.E. St. James Hardy, Tommerup IC, editors; 2001; Albany, Western Australia. pp. 132-137.

10. World Wildlife Fund (2004) Arresting Phytophthora Dieback: The Biological Bulldozer. Perth: World Wildlife Fund.

11. Schirmer J, Field J (2000) The cost of revegetation: Final Report. Canberra, Australia: Australian National University.

12. Arroyo MTK, Marticorena C, Matthei O, Cavieres L (2000) Plant invasions in Chile: present patterns and future predictions. In: Mooney HA, Hobbs RJ, editors. Invasives in a changing world. Washington D.C.: Island Press. pp. 385-421.

13. Pauchard A, Alaback PB (2004) Influence of elevation, land use, and landscape context on patterns of alien plant invasions along roadsides in protected areas of south-central Chile. Conservation Biology 18: 238-248.

14. Forman RTT, Deblinger RD (1998) The ecological road-effect zone of a Massachusetts (USA) suburban highway. Conservation Biology 14: 3-46.

15. Fuentes ER, Jaksic FM, Simonetti JA (1983) European rabbits versus native rodents in Central Chile: effects on shrub seedlings. Oceologia (Berlin) 58: 411-414.

16. Montenegro G, Gomez M, Diaz F, Ginocchio R (2003) Regeneration potential of Chilean Mattorral after fire: an updated view. In: Veblen TT, Baker WL, Montenegro G, Swetnam TW, editors. Fire and climatic change in temperate ecosystems of the Western Americas. New York: Springer-Verlag. pp. 381-409.

17. Montenegro G, Ginocchio R, Segura A, Keely JE, Gomez M (2004) Fire regimes and vegetation responses in two Mediterranean-climate regions. Revista Chilena de Historia Natural 77: 445-464.

18. (2003) Estrategia nacional de biodiversidad. Santiago, Chile: Comisión Nacional del Medio Ambiente.

19. Cowling RM, Lombard AT, Rouget M, Kerley GIH, Wolf T, et al. (2003) A conservation assessment for the Subtropical Thicket Biome. Port Elizabeth: Terrestrial Ecology Research Unit, University of Port Elizabeth.

20. Driver A, Desmet P, Rouget M, Cowling R, Maze K (2003) Succulent Karoo Ecosystem Plan: Biodiversity component. Cape Conservation Unit, Botanical Society of South Africa.

21. Rouget M, Richardson DM, Cowling RM, Lloyd JW, Lombard AT (2003) Current patterns of habitat transformation and future threats to biodiversity in terrestrial ecosystems of the Cape Floristic Region, South Africa. Biological Conservation 112: 63-85.

22. Fairbanks DHK, Thompson MWM, Vink DE, Newby TS, van den Berg HM, et al. (2000) The South African land-cover characteristics database: a synopsis of the landscape. South African Journal of Science 96: 69-82.

23. Rouget M, Reyers B, Jonas Z, Desmet P, Driver A, et al. (2004) South African national spatial biodiversity assessment 2004. Technical report, volume 1 - terrestrial component. Pretoria: South African National Biodiversity Institute.

24. Frazee SR, Cowling RM, Pressey RL, Turpie JK, Lindenberg N (2003) Estimating the costs of conserving a biodiversity hotspot: a case-study of the Cape Floristic Region, South Africa. Biological Conservation 112: 275-290.

25. Landis JD (1998) The Second Generation of the California Urban Futures Model. Parts I, II, and III (with M. Zhang). Environment and Planning, B: Planning and Design 25: 675-666, 795-824.

26. Jones HR (1964) A Bibliographic essay on the urban general plan. In: Kent TJ, editor. The Urban General Plan. pp. 187-210.

27. Yuan-Farrell C, Marvier M, Press D, Kareiva P (2005) Conservation easements as a conservation strategy: is there a sense to the spatial distribution of easements? Natural Areas Journal 25: 282-289.

28. Gustanski JA, Squires RH (2000) Protecting the Land: Conservation Easements Past, Present, and Future. Washington D.C.: Island Press.

29. Bojóquez-Tapia LA, Cueva Hdl, Díaz S, Melgarejo D, Alcantar G, et al. (2005) Environmental conflicts and nature reserves: redesigning sierra San Pedro Mártir Nationa Park, Mexico. Biological Conservation 117: 111-126.

30. Minnich RA (2001) An integrated model of two fire regimes. Conservation Biology 15: 1549-1553.

31. Minnich RA, Chou YH (1996) Wildland fire patch dynamics in the chaparral of southern California and northern Baja California. International Journal of Fire 7: 221-248.

32. California Department of Forestry and Fire Protection (2003) Fire and Resource Assessment Program.

33. FEMA (1995) Federal Emergency Management Agency Flood Zones (Q3).

34. Rundel PW, Sturmer S (1998) Native plant diversity in riparian communities of the Santa Monica Mountains, California. Madroño 45: 93-100.

35. Kuchler AW (1977) Appendix: the map of the natural vegetation of California. In: Barbour MG, Major J, editors. Terrestrial vegetation of California. New York: John Wiley and Sons. pp. 909-938.

36. Brown D, Lowe C (1979) Biotic Communities of the Southwest, Map digitized by The Nature Conservancy in Arizona in 2004.

37. Heaton E, Merenlender AM (2000) Modeling vineyard expansion, potential habitat fragmentation. California Agriculture 54: 12-19.
