# Supplementary material for: Conserving Biodiversity Efficiently: What to Do, Where, and When
Source: PLoS Biol. 2007 Aug 21;5(9):e223. doi: 10.1371/journal.pbio.0050223 (PMC1950771; doi:10.1371/journal.pbio.0050223)
Supplement: Supplementary file 2 [file pbio.0050223.sd002.doc]

**Text S2. Derivation of the parameter alpha**

The tail of the species-area curve beyond the area of conservation interest is an artefact of the construction of the curve, specifically the derivation of the parameter *α*. We generated *α* using the estimated total area of original habitat, not the area of conservation interest. The number of species in each of the three groups constituting the tail of the species-area curve cannot be determined since we do not know the identity of each species or their geographic distributions. Generating the curve in this way provides a more conservative estimate of the likely benefit of each conservation action and species-area curves that are less steep throughout are obtained. Furthermore, it recognises that the persistence of many species depends on past changes to habitat and that our knowledge of species distributions is uncertain. Over 20 years our overall results are insensitive to the two alternative means for deriving *α*. Over shorter timeframes the investment schedules differ slightly, but the general conclusions are robust to changes in *α*.
