# Supplementary material for: Conserving Biodiversity Efficiently: What to Do, Where, and When
Source: PLoS Biol. 2007 Aug 21;5(9):e223. doi: 10.1371/journal.pbio.0050223 (PMC1950771; doi:10.1371/journal.pbio.0050223)
Supplement: Supplementary file 3 [file pbio.0050223.st001.doc]

**Table S1. Data for funding allocation analysis involving only land acquisition**

Table S1 outlines the data employed for a funding allocation analysis that involves only land acquisition. In this analysis, we estimate the cost of land acquisition using a statistical model [1] and the area requiring acquisition as the area of natural habitat that is currently unprotected (IUCN status I-IV). We estimate the biodiversity benefit of this action as the number of plant and vertebrate species in each ecoregion. We determine the area already receiving this action as the area currently protected (IUCN status I-IV).

Table S1. Data employed for a funding allocation analysis that involves only land acquisition.

|  | **Coolgardie woodlands** | **Esperance mallee** | **Eyre and York mallee** | **Jarrah-Karri forest and shrublands** | **Swan Coastal Plain scrub and woodlands** | **Mount Lofty woodlands** | **Murray-Darling woodlands and mallee** | **Naracoorte woodlands** | **Southwest Australia savanna** | **Southwest Australia woodlands** |
| --- | --- | --- | --- | --- | --- | --- | --- | --- | --- | --- |
| **Biodiversity benefit** | 2,143 | 3,281 | 2,383 | 2,487 | 2,658 | 2,393 | 1,600 | 1,874 | 2,357 | 2,338 |
| **Cost per km^2^ (US$)** | 224,787 | 224,787 | 224,787 | 224,787 | 224,787 | 224,787 | 224,787 | 224,787 | 224,787 | 224,787 |
| **Total area km^2^** | 137,226 | 115,460 | 60,896 | 10,455 | 15,210 | 23,786 | 197,895 | 27,531 | 168,713 | 4,6015 |
| **Area requiring action (km^2^)** | 127,038 | 53,706 | 16,015 | 7,366 | 6,747 | 6,157 | 74,492 | 9,317 | 76,258 | 28,645 |
| **Area already receiving action (km^2^)** | 9,166 | 26,503 | 5,776 | 2,898 | 1,325 | 1,622 | 24,055 | 1,509 | 7,028 | 3,120 |

Table S1 cont.

|  | **Chilean matorral** | **Albany thickets** | **Lowland fynbos and renosterveld** | **Montane fynbos and renosterveld** | **Coastal sage scrub and chaparral** | **Interior chaparral and woodlands** | **Montane chaparral and woodlands** |
| --- | --- | --- | --- | --- | --- | --- | --- |
| **Biodiversity benefit** | 2,806 | 1,616 | 3,468 | 6,805 | 2,088 | 2,473 | 2,421 |
| **Cost per km^2^ (US$)** | 170,058 | 184,498 | 184,498 | 184,498 | 829,742 | 1051,174 | 1051,174 |
| **Total area km^2^** | 148,383 | 17,135 | 32,764 | 45,746 | 36,256 | 64,617 | 20,403 |
| **Area requiring action (km^2^)** | 122,934 | 14,567 | 21,050 | 34,352 | 27,713 | 55,528 | 17,594 |
| **Area already receiving action (km^2^)** | 1,332 | 1,403 | 1,183 | 11,027 | 679 | 819 | 2,229 |

**References**

1. Balmford A, Gaston KJ, Blyth S, James A, Kapos V (2003) Global variation in terrestrial conservation costs, conservation benefits, and unmet conservation needs. Proceedings of the National Academy of Sciences of the United States of America 100: 1046-1050.
